# Antibacterial Effect of 445 nm and 970 nm Diode Laser Irradiation in a Simulated Lateral Canal Model: An Ex Vivo Study

**DOI:** 10.3390/bioengineering13101121

**Published:** 2026-09-26

**Authors:** Selma Dervisbegovic, Verena Arnold, Oleh Andrukhov, Alexandra Wolf, Apostolos Georgopoulos, Hassan Shokoohi-Tabrizi, Xiaohui Rausch-Fan, Andreas Moritz, Markus Laky

**Affiliations:** 1 Clinical Division of Periodontology, University Clinic of Dentistry, Medical University of Vienna, 1090 Vienna, Austria; verena.arn99@gmail.com (V.A.); xiaohui.rausch-fan@meduniwien.ac.at (X.R.-F.); andreas.moritz@meduniwien.ac.at (A.M.); markus.laky@meduniwien.ac.at (M.L.); 2 Competence Center for Periodontal Research, University Clinic of Dentistry, Medical University of Vienna, 1090 Vienna, Austria; oleh.andrukhov@meduniwien.ac.at; 3 Core Facility Oral Microbiology and Hygiene, University Clinic of Dentistry, Medical University of Vienna, 1090 Vienna, Austria; alexandra.b.wolf@meduniwien.ac.at (A.W.); apostolos.georgopoulos@meduniwien.ac.at (A.G.); 4 Core Facility Applied Physics, Laser and CAD/CAM-Technology, University Clinic of Dentistry, Medical University of Vienna, 1090 Vienna, Austria; hassan.shokoohi-tabrizi@meduniwien.ac.at; 5 Center for Clinical Research, University Clinic of Dentistry, Medical University of Vienna, 1090 Vienna, Austria

**Keywords:** *Enterococcus faecalis*, diode laser, root canal disinfection, dentin, endodontics, lateral canal, ex vivo model

## Abstract

Persistent *Enterococcus faecalis* infection in inaccessible dentinal regions contributes to endodontic treatment failure, and adjunctive diode laser disinfection has been proposed to reach these areas, but direct comparisons of 445 nm and 970 nm wavelengths in a standardized lateral canal model are lacking. The aim of this study was to evaluate the antibacterial effect of a diode laser through a 0.5 mm dentin barrier in a clinically relevant ex vivo model and to compare its efficacy in reducing *Enterococcus faecalis* at wavelengths of 445 nm and 970 nm. Fifteen extracted human anterior teeth were instrumented to ProTaper F3, embedded in acrylic resin, and prepared with a standardized lateral canal 0.5 mm from the main canal. Following steam sterilization, the canals of specimens were inoculated with *E. faecalis*. Each tooth was exposed to all three conditions (control, 970 nm, 445 nm) across six successive experimental runs, and laser irradiation was performed through root dentin using a SiroLaser Blue diode laser (Dentsply Sirona), yielding 26 run-level measurements per condition overall. For each tooth, log10-transformed CFU/mL values were averaged within each condition prior to statistical analysis, giving one paired value per tooth per condition (*n* = 15). The mean log10-transformed bacterial load was 4.77 ± 0.12 in the control condition, 4.54 ± 0.11 following 970 nm irradiation, and 4.06 ± 0.75 following 445 nm irradiation. The Friedman test showed a significant overall difference among conditions (χ^2^(2) = 15.60, *p* < 0.001; Kendall’s *W* = 0.52), and Holm-adjusted Wilcoxon signed-rank pairwise comparisons (two-sided α = 0.05) showed significantly lower bacterial recovery following both 445 nm (mean tooth-level relative difference 55.9%; adjusted *p* = 0.0013) and 970 nm irradiation (38.2%; adjusted *p* = 0.0013) compared with the control, with significantly lower recovery following 445 nm than 970 nm irradiation (32.1%; adjusted *p* = 0.0215); these relative differences varied considerably among individual teeth. These findings indicate that diode laser irradiation exerts a wavelength-dependent antibacterial effect beyond the directly irradiated canal, supporting its potential as an adjunct to conventional root canal disinfection.

## 1. Introduction

Persistent endodontic infections represent a significant clinical challenge and are one of the main causes of root canal treatment failure requiring retreatment [1,2].

Apical periodontitis, an inflammatory disease often associated with infection of the root canal system, is common and often progresses asymptomatically, with radiographic evidence present in about 39% of root-filled teeth worldwide [3]. Regional data from Austria similarly point to a substantial burden of persistent or postoperative apical disease and to room for improvement in the technical quality and aftercare of endodontic treatment there [4].

Conventional treatment aims to remove infected pulp tissue, disinfect the root canal system, and prevent reinfection through chemomechanical preparation and obturation. However, complete microbial elimination is rarely achieved, as microorganisms can persist within anatomically complex regions—including lateral canals, apical deltas, isthmuses and dentinal tubules—that remain inaccessible to instruments and syringe-delivered irrigants [2,5]. Bacteria have been shown to invade dentinal tubules to depths of approximately 1.0–1.1 mm [6,7], creating a reservoir of infection that may sustain periapical inflammation and contribute to recurrent or persistent apical periodontitis despite technically adequate treatment [1,5,8]. Higher concentrations of sodium hypochlorite (NaOCl) can improve antibacterial efficacy but are associated with an increased risk of tissue irritation, limiting their routine application [2]. Therefore, there is a continued need for adjunctive disinfection strategies capable of reaching deep dentinal sites without compromising safety.

*Enterococcus faecalis* is frequently detected in persistent intraradicular infections and has been reported significantly more often in persistent than in primary endodontic infections [9]. This facultative Gram-positive organism adheres to dentin, forms resilient biofilms, invades dentinal tubules, and can survive nutrient deprivation and conventional antimicrobial measures, properties that make it a clinically relevant target organism in experimental endodontic disinfection models. Its persistence in post-treatment canals is thought to reflect ecological selection under nutrient-poor, antimicrobial-stressed conditions rather than a unique primary pathogenic role, with saliva proposed as a potential reservoir for reinfection of previously accessed or coronally compromised canals [10].

Laser-assisted techniques have been investigated as adjuncts to conventional chemomechanical preparation to enhance disinfection of the root canal system and deeper dentin layers [2,6,7,8]. Erbium-family lasers (Er:YAG, Er,Cr:YSGG), highly absorbed in water and hydroxyapatite, can activate irrigants and improve decontamination of *E. faecalis*-infected canals, particularly when combined with NaOCl or additional laser wavelengths [5,11,12,13]. Nd:YAG irradiation has demonstrated reductions of nearly three log steps in intracanal *E. faecalis* counts in ex vivo models and showed bactericidal effects through 1 mm dentin disks, though complete sterilization was not consistently achieved and mature biofilms remained partially intact [8]. Near-infrared diode lasers, including the 940 and 970 nm wavelengths directly relevant to the present study, have similarly demonstrated reductions in intraradicular bacteria, particularly when combined with chemical irrigants, but diode-only protocols do not reliably sterilize infected canals [7,13,14]. Crucially, experimental dentin-slice models have confirmed that selected wavelengths can exert antibacterial effects through dentin of comparable thickness to clinically relevant infection depths [6,7], supporting the rationale for investigating indirect bactericidal effects beyond the directly irradiated canal surface.

Blue 445 nm diode lasers have increasingly attracted interest over the past decade because their wavelength coincides with absorption peaks of endogenous microbial chromophores, enabling antimicrobial effects via light-induced reactive oxygen species production with comparatively modest temperature increases—suggesting a predominantly photochemical rather than purely thermal mechanism [2,15,16]. In vitro studies have confirmed reductions in viable *E. faecalis* counts under 445 nm irradiation on smooth surfaces [16] and within instrumented root canals [15], while a randomized controlled clinical trial demonstrated that adjunctive 445 nm irradiation further reduced intracanal bacterial load compared with NaOCl irrigation alone [14]. However, Katalinić et al. [15], an in vitro study, did not employ a dedicated lateral canal model, nor did it investigate the indirect bactericidal effect of these wavelengths through root dentin; similarly, the clinical trial by Wenzler et al. [14] did not address this indirect effect through dentin. Comparative data on 970 nm diode irradiation under equivalent experimental conditions also remain limited, with most evidence deriving from studies on other wavelength systems [6,8] or narrative reviews [2]. A direct, side-by-side comparison of 445 nm and 970 nm diode laser irradiation, tested under identical experimental conditions in a standardized lateral canal model and against the same dentin barrier, is therefore still lacking. Closing this specific gap is the central objective of the present study.

The aim of this ex vivo study was to examine the indirect antibacterial effect of diode lasers at 445 nm and 970 nm on *E. faecalis* located across a 0.5 mm dentin barrier, using a standardized lateral canal model in human teeth. The efficacy of both wavelengths was compared against an untreated control group under identical experimental conditions. We hypothesized that both 445 nm and 970 nm diode laser irradiation would reduce *E. faecalis* recovery from the simulated lateral canal compared with the untreated control and that antibacterial efficacy would differ between the two wavelengths.

## 2. Materials and Methods

Prior to the main experiments, a series of preliminary tests was conducted to determine the optimal method for specimen preparation, the appropriate quality and concentration of the inoculum, and the most suitable sampling procedure. Several dilution series were initially prepared to obtain a starting concentration within a countable range capable of demonstrating a clear reduction in bacterial load of approximately one log level. Based on these preliminary results, the final protocol described below was established.

### 2.1. Specimen Preparation

Fifteen extracted human anterior teeth (stored in physiological saline post-extraction) were used. The crowns were removed using a diamond-coated cutting disk, preserving the full root length. Pulp tissue was removed with extirpation needles, and root canals were prepared sequentially with ProTaper hand files (Dentsply Sirona, Charlotte, NC, USA) up to size F3.

Specimens were embedded in silicone molds. Cold-curing acrylic resin (VersoCit-2 Powder, Struers ApS, Ballerup, Denmark) was mixed according to the manufacturer’s instructions, poured into the molds, and allowed to polymerize at room temperature. After complete polymerization, specimens were removed from the molds and trimmed to obtain parallel coronal and apical surfaces. The coronal surface was further smoothed using a polishing machine under continuous water cooling until a uniformly flat surface was achieved.

The drilling site for the lateral canal was defined on the prepared coronal surface. A distance of 0.5 mm from the main root canal was marked using a caliper and verified under a microscope to confirm correct positioning. Specimen blocks were subsequently secured in a holding device to ensure stable processing. At the marked site, a 1.3 mm drill was used to create a vertical hole to a depth of 4 mm. Following drilling, the distance between the lateral canal and the root canal was confirmed under the microscope (Figure 1).

### 2.2. Microbiology

**Inoculum preparation.** *Enterococcus faecalis* strain DSM 20478 was cultured in Brain Heart Infusion (BHI, Becton, Dickinson and Company, Sparks, MD, USA) broth at 37 °C for 24 h. This strain was selected due to its pronounced persistence and frequent detection in endodontic infections, as detailed in the introduction. The bacterial suspension was divided into 150 µL aliquots in sterile cryotubes, supplemented with 15% glycerol as a cryoprotectant, and stored at −80 °C. This approach was used to provide a consistent starting strain and viability across experimental runs.

**Specimen sterilization.** All specimen blocks were sterilized in a tabletop autoclave (CertoClav, CertoClav Sterilizer GmbH, Leonding, Austria) at 121 °C for 20 min. Following sterilization, specimens were stored in sterile physiological saline (0.9% NaCl, B. Braun Melsungen AG, Melsungen, Germany) to prevent dehydration.

**Inoculum dilution and concentration.** From a fresh overnight culture of *Enterococcus faecalis* in BHI, a seven-step serial dilution was prepared as follows: 900 µL of sterile saline (0.9% NaCl, B. Braun Melsungen AG, Melsungen, Germany) was placed into each of seven Eppendorf tubes. Then 100 µL of the bacterial stock suspension was transferred into the first tube and mixed thoroughly; 100 µL was then sequentially transferred through each subsequent tube. From each dilution step, 100 µL was plated onto Columbia agar plates containing 5% sheep blood (bioMérieux Austria GmbH, Vienna, Austria) and incubated at 37 °C for 24 h (BINDER GmbH, Tuttlingen, Germany). Countable colonies (30–300 CFU per plate) were obtained from the 10^−6^ dilution and used to determine the bacterial concentration. Across all dilution series, the mean stock concentration was 2.11 × 10^9^ CFU/mL. For specimen inoculation, the stock solution was diluted 10,000 times, resulting in an inoculum concentration of approximately 2.11 × 10^5^ CFU/mL.

**Specimen inoculation.** Residual saline was removed from each lateral canal by pipetting. Each lateral canal was inoculated with 5 µL of the *E. faecalis* suspension with a concentration of 2.11 × 10^5^ CFU/mL. Specimens were subsequently incubated for 1 h at 37 °C in a humid chamber to allow bacterial adhesion to the canal walls.

### 2.3. Laser Irradiation and Sample Collection

In each experimental run, the 15 specimens were divided into three groups of five, with one group irradiated at 445 nm, one at 970 nm, and one serving as an untreated control. Group assignment rotated across the six successive experimental runs so that, over the course of the study, each of the 15 teeth was tested under all three conditions, yielding a within-tooth crossover design.

Irradiation was performed using a SiroLaser Blue diode laser (Dentsply Sirona, Charlotte, NC, USA) with a 200 µm endodontic laser fiber. Laser power was set to 2 W in continuous-wave mode. The main root canal was irradiated in five intervals of 5 s each, with 20 s intervals between irradiation cycles. Sterile saline was applied uniformly to all root canals during irradiation to prevent dentin dehydration. The control group received no laser treatment (Figure 2).

After treatment, the remaining fluid was pipetted from the lateral canal of each specimen and transferred onto agar plates. Lateral canals were then rinsed with 5 µL of physiological saline to detach residual bacteria from the canal walls; the rinsing fluid was collected and added to the previously transferred fluid on the agar plate. This sampling procedure was performed identically across all groups. After incubation for 24 h at 37 °C, colonies were counted, and the results were converted to CFU/mL.

Because group assignment rotated across runs, each of the 15 teeth contributed measurements to all three conditions, and the experimental procedure was repeated using the same teeth after resterilization. The required sample size (26 measurements per condition) was determined a priori by sample size calculation. Testing was conducted across six experimental rounds (up to five specimens per condition per round, i.e., a maximum of 30 run-level observations per condition); the sixth and final round was therefore only partially completed once this prespecified sample size had been reached, yielding 26 run-level measurements per condition overall.

Between experimental runs, teeth were re-sterilized at 121 °C for 20 min and stored in physiological saline, and a fresh dilution series was prepared to ensure comparable starting conditions and maximize reproducibility.

### 2.4. Statistical Analysis

CFU/mL values were log10-transformed prior to analysis. Because group assignment rotated across the six experimental runs, each of the 15 teeth was tested under all three conditions; for each tooth, the evaluable run-level measurements obtained within a given condition (26 measurements per condition, per the a priori sample size calculation; one or two run-level measurements per tooth; see Section 2.3) were averaged, yielding one paired value per tooth per condition (*n* = 15). This tooth-level averaging avoids treating non-independent, run-level measurements as independent observations. Differences among the three conditions (control, 970 nm, 445 nm) were assessed using the Friedman test on the tooth-level paired means, with Kendall’s W reported as a measure of overall effect size. Pairwise comparisons were performed using two-sided Wilcoxon signed-rank tests on the log10-transformed tooth-level means, with *p*-values adjusted for multiple comparisons using the Holm procedure. Statistical significance was set at a two-sided α of 0.05. Because the 445 nm condition showed marked dispersion, medians and interquartile ranges (IQR) are reported alongside means and SDs. Percentage reductions were calculated for each tooth from the paired arithmetic-mean CFU/mL values and are reported as the mean, with the range across the 15 teeth, rather than implying a uniform effect across specimens. Analyses were performed in Python version 3.14.7 using SciPy 1.17.1. In addition, a post hoc power analysis was performed for a two-sample Wilcoxon Mann–Whitney U test using the online sample size and power calculator provided by the University of Vienna (https://homepage.univie.ac.at/robin.ristl/samplesize.php?test=wilcoxon, accessed on 16 September 2026).

## 3. Results

Bacterial recovery was assessed on a per-tooth basis (*n* = 15); because each tooth was tested under all three conditions across the six experimental runs, repeated measurements obtained from the same tooth within a condition (one or two run-level measurements) were averaged, yielding one paired value per tooth per condition and avoiding pseudoreplication from treating run-level measurements as independent. The mean (±SD) log10-transformed bacterial load was 4.77 ± 0.12 log10(CFU/mL) in the control condition (median 4.79, IQR 4.71–4.87; geometric mean 5.94 × 10^4^ CFU/mL), 4.54 ± 0.11 log10(CFU/mL) following 970 nm irradiation (median 4.56, IQR 4.52–4.61; geometric mean 3.47 × 10^4^ CFU/mL), and 4.06 ± 0.75 log10(CFU/mL) following 445 nm irradiation (median 4.36, IQR 3.90–4.58; geometric mean 1.15 × 10^4^ CFU/mL) (Figure 3). The markedly larger SD and IQR in the 445 nm condition reflect substantial between-tooth heterogeneity in individual response, with some teeth showing near-complete bacterial elimination and others showing little reduction.

The Friedman test showed a significant overall difference among the three conditions (χ^2^(2) = 15.60, *p* < 0.001; Kendall’s *W* = 0.52). In Holm-adjusted Wilcoxon signed-rank pairwise comparisons (two-sided α = 0.05), bacterial recovery was significantly lower following 445 nm irradiation than in the control condition (mean paired difference −0.713 log10 CFU/mL; adjusted *p* = 0.0013), significantly lower following 970 nm irradiation than in the control condition (mean paired difference −0.233 log10 CFU/mL; adjusted *p* = 0.0013), and significantly lower following 445 nm than 970 nm irradiation (mean paired difference −0.480 log10 CFU/mL; adjusted *p* = 0.0215). The mean tooth-level relative differences, calculated for each tooth from paired arithmetic-mean CFU/mL values and averaged across teeth, were 55.9% (range −2.9% to 99.6%) for 445 nm versus control, 38.2% (range −15.9% to 75.2%) for 970 nm versus control, and 32.1% (range −53.2% to 99.1%) for 445 nm versus 970 nm; these relative effects varied substantially among individual teeth, and not every tooth showed a reduction with every wavelength. Individual tooth-level means for each condition, together with the corresponding tooth-level percentage differences, are provided in Supplementary Table S1 to further illustrate this between-tooth variability (see also Figure 3).

A post hoc power analysis using a two-sample Wilcoxon Mann–Whitney U test yielded a power of 0.8693 for control versus 445 nm, 0.9791 for control versus 970 nm, and 0.6124 for 445 nm versus 970 nm. Thus, power exceeded 0.80 for both laser-versus-control comparisons, whereas the direct comparison between the two laser wavelengths showed lower power.

## 4. Discussion

Both 445 nm and 970 nm diode laser irradiation reduced *E. faecalis* recovery from the simulated lateral canal relative to the untreated control, with a stronger effect observed for 445 nm under the tested conditions. This wavelength-dependent pattern suggests that the antibacterial efficacy of diode laser-assisted root canal disinfection is not determined only by the general application of laser energy, but also by the selected wavelength, irradiation parameters, dentin thickness, and the interaction between laser light, dentin, and adherent bacterial cells.

A central aspect of the present study is that bacterial reduction was achieved in an artificial lateral canal located beyond the directly irradiated main canal lumen—anatomy such as lateral canals, dentinal tubules, isthmuses, fins, recesses and apical ramifications that is difficult to reach mechanically or chemically and that previous studies identify as a key reason for persistent infection despite technically adequate treatment [17,18,19,20,21,22]. This supports the concept that diode lasers may accompany and complement, rather than replace, conventional chemomechanical preparation, extending the antibacterial effect of treatment into regions such as simulated lateral canals and deep dentinal structures. This interpretation is consistent with the broader literature describing lasers as adjunctive tools rather than replacements for irrigation and instrumentation [20,23,24,25,26], a view reinforced by studies combining diode lasers with conventional irrigants: diode laser irradiation together with sodium hypochlorite or chlorhexidine has been reported to produce the greatest bacterial reduction, while irrigants alone remained at least as effective as the laser in isolation [24,25,26].

The use of *E. faecalis* as the test organism is appropriate because this microorganism is frequently associated with persistent endodontic infections and is considered difficult to eliminate. Previous studies describe its ability to survive under adverse conditions, form biofilms, invade dentinal tubules and resist conventional intracanal medicaments [17,20,26,27,28,29]. Xu et al. [28] further demonstrated that *E. faecalis* can stimulate inflammatory cytokine expression in macrophage-like cells, supporting its relevance in persistent apical periodontitis. Zou et al. [29] also emphasized the importance of bacterial virulence factors by showing that diode laser irradiation reduced both *E. faecalis* counts and lipoteichoic acid levels. Thus, reducing *E. faecalis* in lateral canals and dentinal structures is not only microbiologically relevant, but may also be important in relation to persistent periradicular inflammation.

The superior antibacterial effect of the 445 nm wavelength observed in the present study should primarily be interpreted as a finding of the present experimental model. It is consistent with the broader concept that diode laser efficacy is wavelength- and parameter-dependent, but it is not uniformly demonstrated across all previous studies. Hoshyari et al. [27] compared 445 nm and 970 nm diode laser irradiation, photodynamic therapy and triple antibiotic paste against *E. faecalis* in root canals. In that study, both diode laser wavelengths showed antibacterial activity, although the strongest effects were observed in the triple antibiotic paste and photodynamic therapy groups. Hendi et al. [17] investigated a 445 nm diode laser and an Er,Cr:YSGG laser in dentin slices of different thicknesses. Their results showed that dentin thickness influenced the antibacterial effect; however, neither the 445 nm diode laser nor the Er,Cr:YSGG laser alone significantly reduced bacterial counts across the tested thicknesses. A significant reduction was observed only in 1 mm dentin sections when the Er,Cr:YSGG and 445 nm diode lasers were combined. Therefore, these studies support the antibacterial potential and parameter dependence of blue diode laser application, but they also show that 445 nm laser irradiation alone may not be consistently effective under all experimental conditions.

The significant effect of the 970 nm diode laser in the present study (38.2% reduction relative to control; *p* < 0.001) is also consistent with previous investigations of near-infrared diode lasers. Tanner et al. [20] showed that 970 nm diode laser irradiation reduced the viability of mature *E. faecalis* biofilms in single-rooted teeth, with continuous-wave parameters producing greater bacterial reduction than pulsed modes. Romeo et al. [30] reported antibacterial effects of a 980 nm diode laser against *E. faecalis* biofilm in experimentally infected root canals, particularly when combined with conventional chemomechanical treatment. Pelozo et al. [31] also showed that 980 nm diode laser irradiation reduced total microbial counts and *E. faecalis* counts during retreatment, particularly before complete biomechanical preparation. Therefore, the present 970 nm results are supported by previous evidence, although the significantly stronger performance of 445 nm in the present study indicates that the two wavelengths should not be considered equivalent under the tested conditions.

The mechanism responsible for the difference between 445 nm and 970 nm cannot be definitively explained by the present study because temperature development, dentin optical properties and bacterial structural changes were not directly assessed. However, the previous literature suggests that different wavelengths may act through different physical and biological mechanisms. Pirnat et al. [32] showed that near-infrared Nd:YAG and diode laser irradiation had little direct bactericidal effect on non-pigmented *E. faecalis* when substrate heating was excluded, indicating that the bacterial microenvironment and photothermal interactions may be important for near-infrared lasers. Tanner et al. [20] also interpreted the antibacterial effect of 970 nm diode irradiation mainly in relation to photothermal activity while emphasizing the need to control temperature rise. In contrast, visible blue wavelengths may interact differently with bacterial structures or chromophores, but this cannot be confirmed from the present data. Therefore, the greater efficacy of 445 nm should be described as a wavelength-specific experimental finding rather than as proof of a defined mechanism.

Thermal safety is an important consideration when interpreting diode laser disinfection. Tanner et al. [20] showed that higher-power 970 nm continuous irradiation produced strong antibacterial effects but also caused slight carbonization inside the root canal. Zou et al. [29] found that higher diode laser power produced greater bacterial and LTA reduction, but that 2.5 W exceeded the safe thermal threshold in their model. These findings highlight the need to balance antibacterial efficacy with avoidance of thermal damage to dentin and periodontal tissues. In the present study, both wavelengths reduced bacterial load, but future studies should directly evaluate temperature changes at the external root surface and within dentin during continuous-wave irradiation at both wavelengths to determine the safest and most effective clinical parameters.

The present findings should also be distinguished from studies on laser-activated irrigation. Laser-activated irrigation primarily depends on irrigant activation, cavitation, bubble dynamics and improved fluid movement, whereas the present study evaluated diode laser irradiation as an antibacterial adjunct acting through dentin toward a lateral canal. Swimberghe et al. [18] demonstrated that laser-activated irrigation acts through laser-induced cavitation bubbles and rapid movement of canal contents, which can dislodge biofilms and transport them coronally. These findings should therefore be interpreted as evidence for a hydrodynamic cleaning mechanism rather than as direct evidence of diode laser antibacterial penetration. Similarly, Meire and De Moor [19] described the principles and antimicrobial potential of laser-activated irrigation, but this approach differs from the direct diode laser irradiation model used in the present study. Wang et al. [33] found that Er:YAG- and Er,Cr:YSGG-activated sodium hypochlorite achieved stronger bacterial reduction in dentinal tubules than diode laser irradiation alone. Abaza et al. [21] also reported superior smear layer removal with Er,Cr:YSGG laser activation compared with diode laser activation, passive ultrasonic irrigation and conventional syringe irrigation. These studies are important for contextualization, but they do not directly contradict the present findings because the present study focused on wavelength-specific diode laser effects in a lateral canal model rather than on irrigant activation.

Photodynamic approaches provide another relevant comparison. Morikawa et al. [34] demonstrated that photodynamic therapy using an Nd:YAG laser with resveratrol and pigment reduced *E. faecalis*, with complete absence of colonies in the group combining resveratrol, red pigment and laser irradiation. Hoshyari et al. [27] also reported that photodynamic therapy with doxycycline produced significant bacterial reduction. Al-Jaberi et al. [22] compared photoactivated disinfection and conventional laser endodontics and found that diode laser-assisted protocols improved bacterial reduction compared with conventional irrigation alone, with the triple-wavelength group producing the lowest colony-forming units. Together, these studies demonstrate that laser-assisted disinfection can operate through different mechanisms, including direct photothermal effects, photodynamic effects and irrigant activation. The present study contributes specifically by showing that diode laser irradiation alone can reduce bacteria in a simulated lateral canal and that this effect differs between 445 nm and 970 nm.

Clinical studies among the previous literature also support the broader relevance of diode lasers in endodontics. Kaplan et al. [35] found that 980 nm diode laser irradiation after conventional irrigation reduced postoperative pain in teeth with apical periodontitis. Ismail et al. [36] reported that low-level laser therapy and laser-activated irrigation reduced postoperative pain compared with mock laser treatment, particularly during the first 24–48 h. Pelozo et al. [31] found that 980 nm diode laser irradiation improved periapical healing during follow-up, even though its additional microbiological effect after full retreatment was not superior to conventional preparation alone. Kadam et al. [37], in a systematic review, concluded that photobiomodulation may be beneficial as an adjunct for postoperative pain management, although heterogeneity in protocols prevents firm recommendations. In line with this, 980 nm irradiation under clinically relevant low-level laser therapy conditions has also been shown to exert anti-inflammatory rather than purely antimicrobial effects on periodontal host cells [38], suggesting that near-infrared diode wavelengths may contribute to tissue healing through mechanisms beyond direct bacterial killing. Although these clinical studies primarily address pain and healing rather than lateral canal disinfection, they support the clinical interest in laser-assisted endodontic adjuncts.

The marked between-tooth heterogeneity observed in the 445 nm condition also warrants comment. Plausible contributors include differences in dentin density or mineralization, dentinal tubule density and orientation, local dentin thickness, sclerosis, and micro-cracks, any of which could alter the scattering, absorption, and transmission of laser light through dentin, as well as possible variation in bacterial adhesion or recovery between specimens. None of these structural or optical properties were measured in the present study, so they should be regarded as plausible explanations rather than demonstrated mechanisms for the observed variability. Future studies combining microbiological outcomes with structural or optical characterization of individual teeth would help clarify the sources of this heterogeneity.

Several methodological strengths of the present study should be emphasized. The artificial lateral canal model allowed the antibacterial effect to be tested in a standardized side canal region, which directly reflects the clinical challenge of disinfecting inaccessible anatomy. Group assignment rotated across experimental runs so that each tooth served as its own control across conditions, and this within-tooth paired structure was accounted for using the Friedman test with Holm-adjusted Wilcoxon signed-rank pairwise comparisons on tooth-level means, avoiding pseudoreplication of run-level measurements. In addition, the use of *E. faecalis* provided a resistant and clinically relevant test organism. The finding that both wavelengths significantly reduced bacterial recovery from the lateral canal, with a significantly stronger effect for 445 nm, therefore provides experimental support for wavelength-specific diode laser-assisted disinfection in a lateral canal model, although the considerably greater dispersion observed in the 445 nm condition indicates that individual tooth-level responses to this wavelength were less uniform than the mean effect suggests.

Nevertheless, some limitations must be considered. First, the study was conducted ex vivo using extracted teeth. Although this allowed strict standardization, it cannot fully reproduce the clinical environment, including periodontal blood flow, tissue fluid, immune responses and complex root canal microbiota. Second, the study used a single-species *E. faecalis* model. Clinical root canal infections are polymicrobial, and multispecies biofilms may respond differently to laser irradiation. Third, culture-based methods quantify cultivable bacteria but may fail to detect viable but non-culturable cells. Wang et al. [33] and Tanner et al. [20] used fluorescence-based viability methods, which may provide additional information about bacterial survival and membrane integrity. Fourth, only one irradiation protocol was evaluated for each wavelength. Since the previous literature shows that laser power, irradiation time, emission mode, fiber diameter, fiber movement, dentin thickness and biofilm maturity can all influence antibacterial efficacy, further parameter testing is necessary [17,19,20,29]. Fifth, the number of repeated measurements per tooth and condition was not perfectly balanced (one or two run-level measurements per tooth and condition); tooth-level averaging avoids pseudoreplication but gives equal weight to teeth with different numbers of underlying measurements. An exploratory linear mixed-effects model (condition and experimental run as fixed effects, tooth as a random intercept) was attempted as a sensitivity check but did not converge to a stable, well-identified solution (the tooth-level random-effects variance was estimated at or near the boundary), so its estimates are not reported here; a fully specified, converged mixed-effects analysis is left for future work with a larger and more balanced dataset. Sixth, the tooth-level relative reductions varied considerably between specimens (e.g., −2.9% to 99.6% for 445 nm versus control), and the mean relative effect should not be interpreted as uniform across individual teeth. Seventh, the same specimens were reused across experimental runs and re-sterilized by autoclaving between rounds; repeated steam sterilization may have altered dentin structure, physicochemical properties, or the optical transmission and scattering of laser energy through dentin, but these properties were not measured in the present study, so the direction or magnitude of any such effect cannot be determined.

## 5. Conclusions

Within these limitations, the present study indicates that diode laser irradiation may reduce *E. faecalis* in a simulated lateral canal and may support the disinfection of anatomical areas not predictably reached by conventional preparation. The stronger effect of the 445 nm laser suggests that blue diode laser irradiation may offer an advantage over 970 nm irradiation under the tested conditions; however, this remains to be confirmed under different protocols and in clinical scenarios. Because neither wavelength achieved complete bacterial elimination, diode laser irradiation should be regarded as an adjunctive strategy that accompanies conventional chemomechanical treatment rather than replacing it. Future studies should evaluate multispecies biofilms, different lateral canal diameters and distances, additional dentin thicknesses, thermal safety, and combined protocols with irrigants in order to clarify the clinical relevance of wavelength-specific diode laser disinfection.

## Figures and Tables

**Figure 1:**
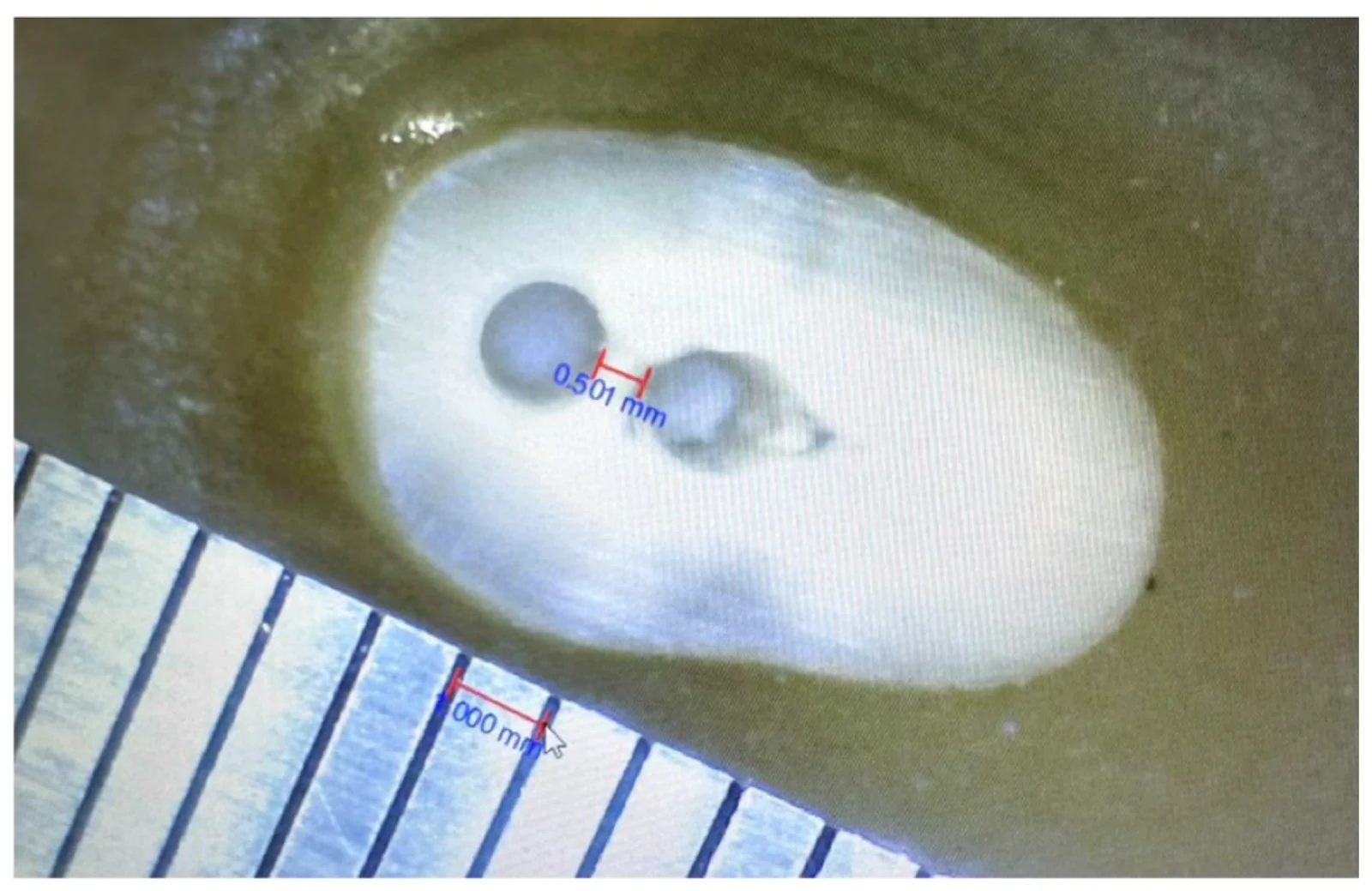
Specimen after drilling of the lateral canal, viewed under the microscope to confirm the distance between the lateral canal and the main root canal (0.501 mm).

**Figure 2:**
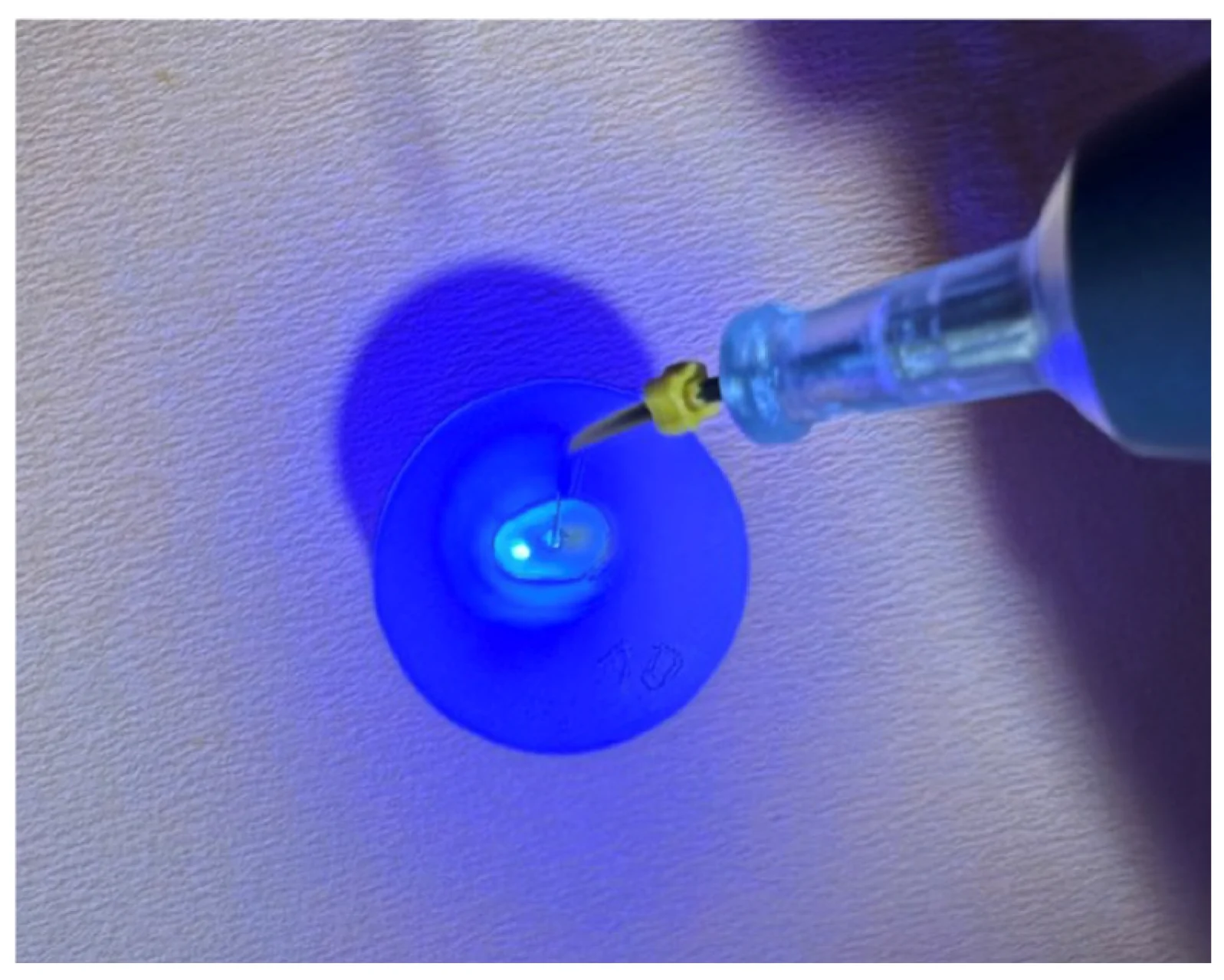
Laser irradiation of a specimen (445 nm shown), with the endodontic laser fiber positioned in the main root canal.

**Figure 3:**
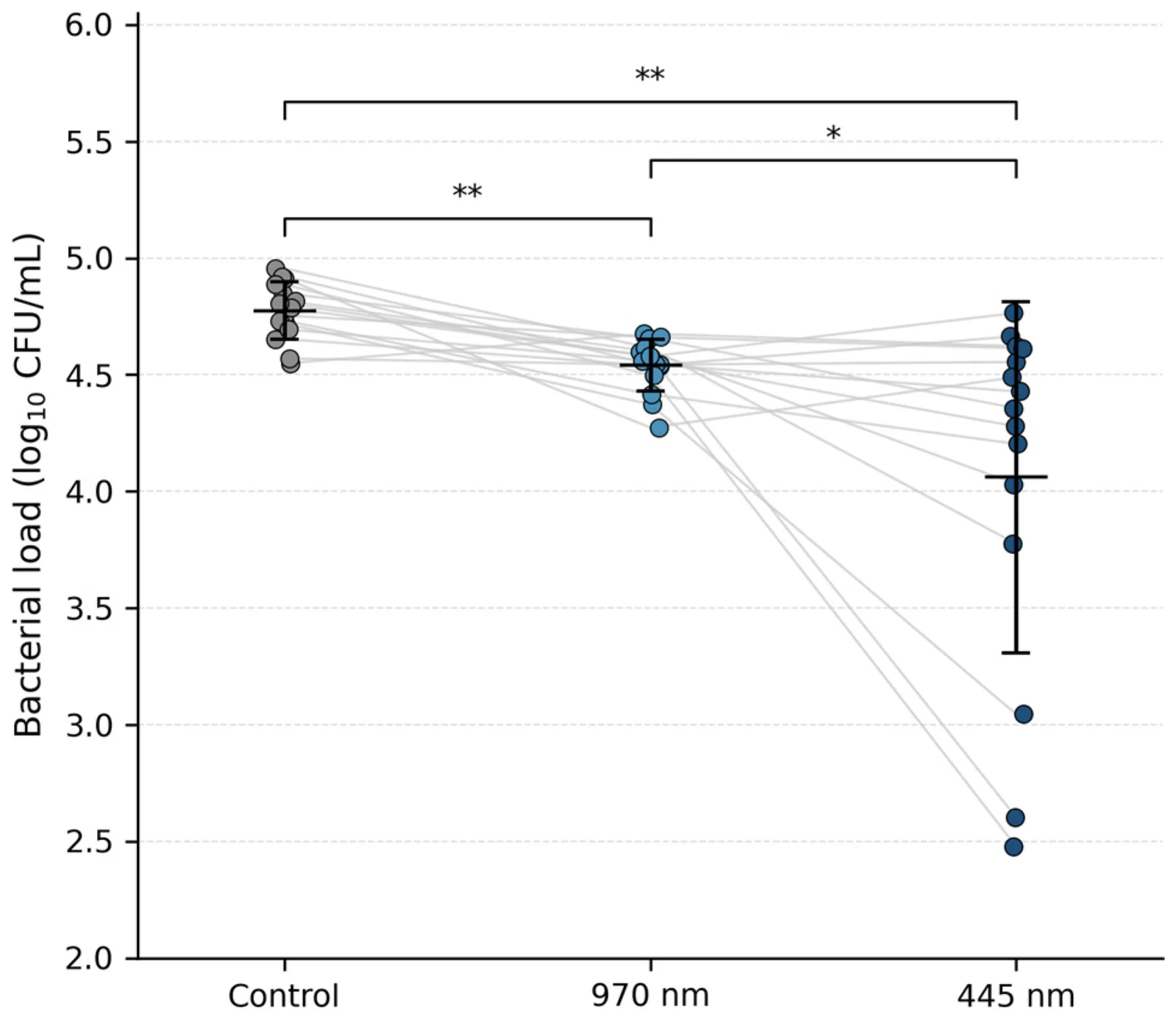
Bacterial load (log_10_ CFU/mL) in the control, 970 nm, and 445 nm conditions, shown as one paired mean value per tooth (*n* = 15) per condition; each point represents the mean of one or two log10-transformed run-level measurements for that tooth and condition. Gray lines connect values from the same tooth across conditions; horizontal bars indicate the mean ± SD. Asterisks denote significant Holm-adjusted Wilcoxon signed-rank pairwise comparisons (two-sided α = 0.05): single asterisk, adjusted *p* < 0.05; double asterisk, adjusted *p* < 0.01.

## Data Availability

The data presented in this study are available on request from the corresponding author.

## Supplementary Materials

The following supporting information can be downloaded at: https://www.mdpi.com/article/10.3390/bioengineering13101121/s1, Table S1: Individual tooth-level mean bacterial recovery (CFU/mL) for the control, 970 nm, and 445 nm conditions, and the corresponding tooth-level percentage differences (*n* = 15 teeth).

## Abbreviations

The following abbreviations are used in this manuscript:

BHI	Brain Heart Infusion
CFU	Colony-Forming Unit
NaOCl	Sodium Hypochlorite
