# Supplementary material for: Antibacterial Effect of 445 nm and 970 nm Diode Laser Irradiation in a Simulated Lateral Canal Model: An Ex Vivo Study
Source: Bioengineering (Basel). 2026 Sep 26;13(10):1121. doi: 10.3390/bioengineering13101121 (PMC13701882; doi:10.3390/bioengineering13101121)
Supplement: Supplementary file 1 [file bioengineering-13-01121-s001.zip › bioengineering-4529005-supplementary.pdf]

Table S1. Individual tooth-level mean bacterial recovery (CFU/mL) for the control, 970-nm, and 445-nm conditions, and the corresponding tooth-level percentage differences (n = 15 teeth).

Tooth	Control Mean (CFU/mL)	970-nm Mean (CFU/mL)	445-nm Mean (CFU/mL)	% Diff 445 vs. Control	% Diff 970 vs. Control	% Diff 445 vs. 970
1	48,500	35,700	46,600	3.9	26.4	−30.5
2	56,700	46,050	41,300	27.2	18.8	10.3
3	90,200	41,450	6150	93.2	54.0	85.2
4	53,900	26,750	16,000	70.3	50.4	40.2
5	82,000	20,300	31,100	62.1	75.2	−53.2
6	50,450	38,700	20,850	58.7	23.3	46.1
7	63,900	38,150	58,200	8.9	40.3	−52.6
8	83,700	35,050	26,800	68.0	58.1	23.5
9	76,900	32,800	300	99.6	57.3	99.1
10	64,950	38,650	400	99.4	40.5	99.0
11	40,900	47,400	42,100	−2.9	−15.9	11.2
12	71,100	45,200	22,750	68.0	36.4	49.7
13	39,250	34,900	38,350	2.3	11.1	−9.9
14	65,750	39,700	10,750	83.7	39.6	72.9
15	54,950	23,600	2200	96.0	57.1	90.7
